# Towards precision psychiatry utilizing large-scale multimodal data paving the way for improved prevention and treatment of mental disorders

**DOI:** 10.1016/j.nsa.2022.101017

**Published:** 2022-12-22

**Authors:** Jonas Meisner, Simon Rasmussen, Michael E. Benros

**Affiliations:** a Biological and Precision Psychiatry, Copenhagen Research Centre for Mental Health, Mental Health Centre Copenhagen, Copenhagen University Hospital, Denmark; b Novo Nordisk Foundation Center for Protein Research, Faculty of Health and Medical Sciences, University of Copenhagen, Denmark; c Department of Immunology and Microbiology, Faculty of Health and Medical Sciences, University of Copenhagen, Denmark

**Keywords:** Precision psychiatry, Machine learning, Deep learning, Multimodal data

Meyer-Lindenberg and colleagues (Meyer-Lindenberg et al., 2022) highlight how new opportunities in research and treatment strategies can advance neuroscience and related fields by focusing on refined diagnostics, prevention, and early intervention. Treatment strategies for mental disorders are based on imprecise “one-size-fits-all” therapies based on the current broad diagnostic entities. However, the mental disorders are heterogeneous and likely have a complex etiology with both environmental and genetic underpinnings. Psychiatry is therefore in need of new tools and insights to stratify patients into more homogeneous groups to provide better treatment including targeted drug interventions. Precision psychiatry is a rapidly developing field focusing on predictive modeling of mental disorders to develop more precise treatment strategies through stratification of the current mental disorder entities and trajectories, but it is struggling to make a major clinical impact due to a limited scope in clinical utility and robustness for the developed prediction models (Meehan et al., 2022). However, precision psychiatry approaches hold great promises when focusing on improved diagnostics and prevention through utilizing large-scale data and novel methods needed to unravel the complexity of mental disorders, such as artificial intelligence (Meyer-Lindenberg et al., 2022), while seeking to prioritize clinical relevance and validation of models across cohorts (Meehan et al., 2022). This could help to gain new insights into mental disorders and improve current treatment approaches.

The increasing wealth of big health data, as is present in Denmark, in the form of national register data, large-scale genetic data, population-based electronic health records (EHRs) with clinical notes and brain scans, provides opportunities to utilize state-of-the-art deep learning approaches that can integrate and combine the different multimodal data sources. Machine learning models have already demonstrated their ability to identify distinct disease trajectories of patients with mental disorders based on high-dimensional national register data combined with large genetic cohorts (Krebs et al., 2021; Allesøe et al., 2022a, 2022b). Deep learning has in the recent decade transformed the fields of natural language processing and image analysis, and it has already made tremendous impact in the life sciences, thus demonstrating the power and flexibility of the developed models (LeCun et al., 2015). The advantage of deep learning is its ability to model high-dimensional data as well as jointly model multimodal data to learn non-linear complex relationships not possible with standard methods. The promising performance in natural language processing and image analysis will be highly relevant for the processing of free text in clinical notes from EHRs and brain imaging for mental disorders. Utilizing machine learning approaches EHR data have already been leveraged to identify individuals at high risk of acute readmission (Rajkomar et al., 2018). Combining the predictive power of deep learning models with the large-scale data available, it will be possible to disentangle the complexity of mental disorders at a scale and resolution that has not previously been possible using traditional prediction models and will introduce new opportunities for personalized treatment strategies.

Currently, brain scans are mainly used in clinical psychiatry to rule out evident organic causes for symptoms. Nonetheless, brain imaging of individuals with mental disorders have been of great interest in psychiatric research but recent studies have shown the need for large-scale brain imaging datasets to find reliable associations related to mental disorders due to the high statistical noise (Marek et al., 2022). However, with the EHRs in Denmark, researchers will have access to large-scale datasets where biomedical segmentation models (Ronneberger et al., 2015), based on convolutional neural networks (LeCun et al., 2015), can be used to extract image-derived phenotypes and objective biomarkers from the brain scans. Brain scans therefore have great potential to increase our understanding of biological underpinnings to mental disorders, as we can discover novel neuroimaging patterns related to severity and disease trajectory.

Of relevance to the use of clinical notes in the EHR, the deep learning transformer architecture for sequence modeling has allowed for parallel processing of texts, which has led to a substantial performance gain in natural language processing (Vaswani et al., 2017). The transformers have already been applied on clinical notes from EHRs to learn deep representations and have shown superior performance for predicting acute hospital readmissions (Huang et al., 2019). The transformer architecture models long-range temporal dependencies that can be used to model disease diagnose patterns in the clinical notes and learn individual representations. These representations capture disease trajectories and can be used for patient stratification to improve individual treatment strategy including targeted drug interventions as well as be linked with structured EHR data and brain scans for improved diagnostics. The patient stratification from the brain scans, clinical notes or their combined effort will also provide objective data-driven phenotypes that when linked with genetic data can be used for association studies to reveal novel genetic underpinnings.

However, even with the demonstrated predictive power of current machine learning applications in precision psychiatry and medicine, their full clinical impact has yet to be uncovered. Main limitations are often the use of data that may not generalize to the full population, introducing sampling bias, as well as having limited scope in regards to clinical validation. Researchers need to develop models that can be clinically implemented in different environments with emphasis on interpretability to validate the clinical validity of the predictions from the models (Vokinger et al., 2021). Access to a population-based EHR platform in the hospitals holds the advantage of covering the full population and offering direct implementation of applications in clinics for immediate clinical validation that will lead to robust and generalizable models, while mitigating for potential bias.

Machine and deep learning approaches will likely have a major impact towards precision psychiatry, where the integration of multiple large-scale data sources will result in a paradigmatic shift and help disentangling the complex etiology of mental disorders. The different modalities will help enlighten the complexity of mental disorders from multiple angles that jointly will increase the predictive power and even illuminate complex relationships between them. However, feasible clinical implementation must be a focus and emphasized early in the model development as the employment of these models into EHR platforms will be key to pave the way for improved diagnostics, prevention and treatment of mental disorders and has not been possible with prior models. EHRs therefore present an unique opportunity in which models can be rapidly validated and replicated in existing clinical systems by direct implementation in consultation with clinicians. Successful development and implementation of machine learning models could lead to better personalized treatment through the superior predictive performance of the models and their opportunity to aid clinicians as decision support tools.
